# Neuroticism and adverse life events are important determinants in functional somatic disorders: the DanFunD study

**DOI:** 10.1038/s41598-022-24213-6

**Published:** 2022-11-15

**Authors:** Marie Weinreich Petersen, Tina Birgitte Wisbech Carstensen, Lisbeth Frostholm, Kaare Bro Wellnitz, Eva Ørnbøl, Thomas Tandrup Lamm, Thomas Meinertz Dantoft, Lene Falgaard Eplov, Torben Jørgensen, Per Fink

**Affiliations:** 1 Research Clinic for Functional Disorders and Psychosomatics, Aarhus University Hospital, Palle Juul Jensens Boulevard 11, 8200 Aarhus N, Denmark; 2 Department of Clinical Medicine, University of Aarhus, Aarhus, Denmark; 3 Center for Clinical Research and Prevention, Bispebjerg and Frederiksberg Hospital, The Capital Region of Denmark, Copenhagen, Denmark; 4 Copenhagen Research Center for Mental Health, Mental Health Centre Copenhagen, The Capital Region of Denmark, Copenhagen, Denmark; 5 Department of Public Health, Faculty of Health and Medical Science, University of Copenhagen, Copenhagen, Denmark

**Keywords:** Medical research, Risk factors

## Abstract

Several psychological factors have been proposed to be associated with functional somatic disorders (FSD). However, large population-based studies investigating the importance of both personality and adverse life events (ALE) are sparse. This study aimed to investigate the association between FSD and neuroticism and the accumulated number of ALE, respectively. This cross-sectional study included a random sample of the adult Danish population (*N* = 7493). FSD were established by means of self-reported questionnaires and diagnostic interviews. Neuroticism was measured with the Danish version of the short-form NEO Personality Inventory. ALE were measured with the Danish version of the Cumulative Lifetime Adversity Measure. Strong positive associations were found between neuroticism and FSD, and ALE and questionnaire-based FSD. For interview-based FSD, strong positive associations were found for FSD, multi-organ type, and for the subtype of the general symptoms. The level of self-efficacy did not modify these associations, and no moderating effect of neuroticism and ALE in combination on the probability of having FSD was found. FSD were strongly associated with both neuroticism and the accumulated number of ALE, and these associations were not modified by self-efficacy. In combination, neuroticism and ALE did not have a moderating effect on the probability of having FSD.

## Introduction

Functional somatic disorders (FSD) are conditions characterized by persistent patterns of impairing physical symptoms which cannot be better explained by other physical or mental conditions^1^. FSD is a unifying diagnosis which includes functional somatic syndromes (FSS) such as irritable bowel (IB), chronic widespread pain (CWP), and chronic fatigue (CF)^2^. Patients with FSD may be severely disabled and emotionally distressed, and patients with severe cases have an excessive use of healthcare services in terms of repeated hospitalizations, medical investigations, and fruitless treatment attempts^1,3^. Thus, FSD are costly for patients and society, both as to healthcare costs, lost working years, early retirement, and other social expenses.

The aetiology of the conditions is considered multifactorial, i.e. involving biological, psychological, and social factors^1,4,5^. Several psychological factors such as personality and exposure to trauma/adverse life events have been suggested to be involved in the onset and perpetuation of FSD.

Clinical studies have shown neuroticism (i.e. the tendency to experience negative emotions and cognitions^6^) to be pronounced in patients with chronic fatigue syndrome^7–10^, multi-organ bodily distress syndrome^11^, somatoform disorders^12^, and dissociative disorders^13^. However, a study by Kingma et al.^14^ performed in a representative community-based population cohort, establishing FSD by means of participant’s self-report from a pre-defined list of FSD diagnoses, did not find an association between FSD and neuroticism.

Some population-based studies have found strong associations between adverse life events (ALE) and functional somatic syndromes^15,16^ and functional somatic symptoms^17^. Lastly, a meta-analysis including 71 case–control studies concluded that individuals having experienced ALE had a higher risk of having a functional somatic syndrome^18^. One clinical study including patients with dissociative disorders investigated both personality traits and ALE and found associations between dissociative disorders and neuroticism and emotional, physical, and sexual abuse^13^.

The level of self-efficacy determines the cognitive appraisal of stressful situations, e.g. induced by the experience of ALE^19,20^, and it has been shown that self-efficacy correlates negatively with negative affect, i.e. neuroticism^21^. A population-based study has found self-efficacy to mediate the association between chronic stress and personality trait^22^. Therefore, self-efficacy may be an important factor to consider in the analyses of the relationship between FSD, neuroticism, and ALE.

Most knowledge of the association between FSD and the various psychological factors has until now been poorly supported by data from small, cross-sectional studies in highly selected patient samples. This may induce a great risk of selection and publication bias. Therefore, in order to reinforce the established theories on this field, highly powered cohort studies including unselected population-based samples are needed to establish the association between the various psychological factors and FSD.

The present study included a large unselected sample from the adult Danish population. The objectives were (1) to estimate the association between FSD and neuroticism and investigate if this association was modified by the level of self-efficacy, (2) to estimate the association between FSD and the accumulated number of ALE and investigate if this association was modified by the level of self-efficacy, and (3) to investigate the possible interaction between neuroticism and the accumulated number of ALE in the association with FSD.

The following hypotheses were made:

1. A higher score on neuroticism is associated with FSD, and this association is modified by self-efficacy, i.e. higher self-efficacy reduces the association between neuroticism and FSD.
2. Experiencing more ALE is associated with FSD, and this association is modified by self-efficacy, i.e. higher self-efficacy reduces the association between ALE and FSD.
3. Higher scores on neuroticism and experiencing more ALE further increase the association with FSD.

## Results

### Characteristics of study participants

Median age of the 7493 participants that filled in self-reported symptom questionnaires was 54 years (IQR 44–64), and 53.9% were women. Mean score of neuroticism was 16.3 (SD = 7.4); median number of accumulated ALE was 5 (IQR 3–8), and median score on self-efficacy was 21 (IQR 18–25).

Median age of the stratified subsample of 1590 participants that took part in the diagnostic research interview was 54 years (IQR 44–63); 59.3% were women. Mean score of neuroticism was 18.7 (SD = 8.4); median number of accumulated ALE was 6 (IQR 4–8), and median score on self-efficacy was 21 (IQR 17–25).

### Functional somatic disorders and the association with neuroticism

Strong positive associations between neuroticism and FSD were found, regardless of how the FSDs were defined and assessed. In none of the analyses could we reject the hypothesis of no modification by level of self-efficacy. Table 1 shows the odds ratio of having FSD when comparing two individuals differing only one point on neuroticism. The association with neuroticism was especially strong for the multi-organ type of FSD. Regarding the single-organ subtypes of FSD, the musculoskeletal subtype stood out as having the weakest, however, still significant association with neuroticism. Regarding the three functional somatic syndromes, CF stood out as having the strongest association with neuroticism and this remained in the pure type of CF without comorbidity of other functional somatic syndromes.

**Table 1:** Association between functional somatic disorders and neuroticism.

	Questionnaire-based	Interview-based
	OR (95% CI)	OR (95% CI)
**Overall functional somatic disorder (Q *n* cases = 1220; I *n* cases = 394)**	1.08 (1.07–1.10)	1.08 (1.06–1.10)
**Single-organ type (Q *n* cases = 1141; I *n* cases = 311)**	1.08 (1.07–1.09)	1.07 (1.05–1.09)
Cardiopulmonary (Q *n* cases = 71; I *n* cases = 46)	*1.14 (1.10–1.19)*^1^	*1.12 (1.07–1.16)*^2^
Gastrointestinal (Q *n* cases = 279; I *n* cases = 158)	*1.10 (1.08–1.12)*	*1.09 (1.06–1.11)*
Musculoskeletal (Q *n* cases = 733; I *n* cases = 102)	*1.06 (1.05–1.07)*	*1.04 (1.01–1.07)*^2^
General symptoms/fatigue (Q *n* cases = 265; I *n* cases = 109)	*1.15 (1.13–1.18)*	*1.11 (1.08–1.14)*^2^
**Multi-organ type (Q *n* cases = 79; I *n* cases = 83)**	1.20 (1.16–1.23)^3^	1.11 (1.07–1.15)^3^
**Irritable bowel (*n* cases = 257)**	1.10 (1.08–1.12)	–
Irritable bowel, pure type (*n* cases = 144)	*1.06 (1.03–1.09)*	–
**Chronic widespread pain** (*n* cases = 325)	1.08 (1.06–1.10)	–
Chronic widespread pain, pure type (*n* cases = 176)	*1.04 (1.01–1.06)*	–
**Chronic fatigue (*n* cases = 658)**	1.12 (1.11–1.14)	–
Chronic fatigue, pure type (*n* cases = 464)	*1.11 (1.10–1.13)*	–

Odds ratio of having functional somatic disorder comparing two individuals who differ one point on neuroticism.

Adjusted for sex, age, social status, accumulated number of adverse life events, and self-efficacy.

In all analyses, *p* < 0.05.

*Q* questionnaires, *I* interview.

^1^Only adjusted for sex, age, social status, and accumulated number of adverse life events.

^2^Only adjusted for sex, age, and social status.

^3^Only adjusted for sex and age.

Significant values are in italics.

### Functional somatic disorders and the association with the accumulated number of adverse life events

The effect of the accumulated number of ALE on FSD did not fulfil the assumptions of linearity. Therefore, the associations were illustrated with restricted cubic splines. Having experienced more than 15 ALE was rare (only 1.5% of the total sample). Hence, only results restricted to a maximum of 15 ALE were shown. The median number of accumulated ALE was used as reference value.

**Figure 1:**
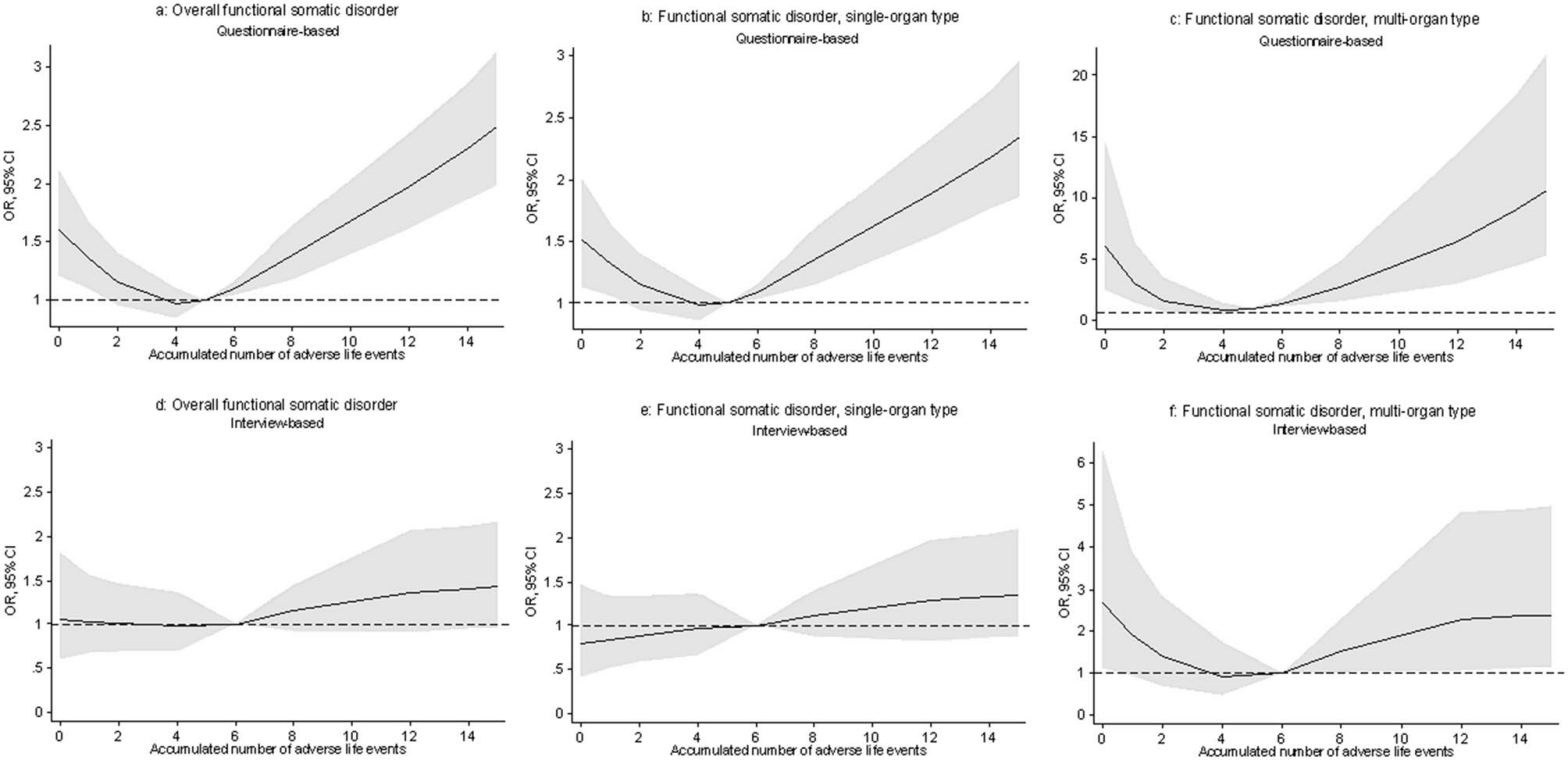
Association between the accumulated number of adverse life events and functional somatic disorders. Cases were established by means of both self-reported questionnaires and diagnostic interviews. (**a,b,d,e**) were adjusted for sex, age, social status, personality, and self-efficacy. (**c,f**) Were only adjusted for sex because of low number of cases. NB! The OR scales for the multi-organ types (**c,f**) have different ranges than the OR scales for the other types.

**Figure 2:**
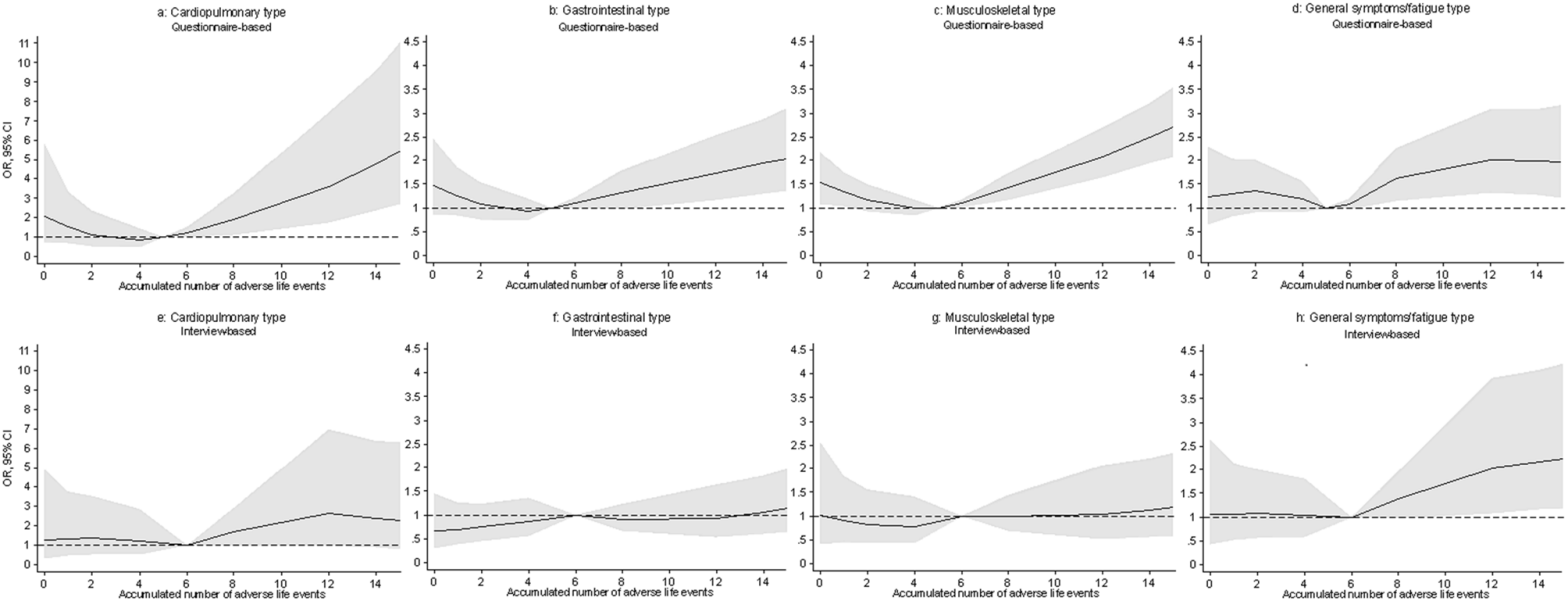
Association between the accumulated number of adverse life events and single-organ subtypes of functional somatic disorders. Cases were established by means of both self-reported questionnaires and diagnostic interviews. (**a**) Was adjusted for sex and age; (**e**) was not adjusted; (**b–d,f–h**) were adjusted for sex, age, social status, personality, and self-efficacy. NB! The OR scales for the cardiopulmonary types (**a,e**) have different ranges than the OR scales for the other types.

**Figure 3:**
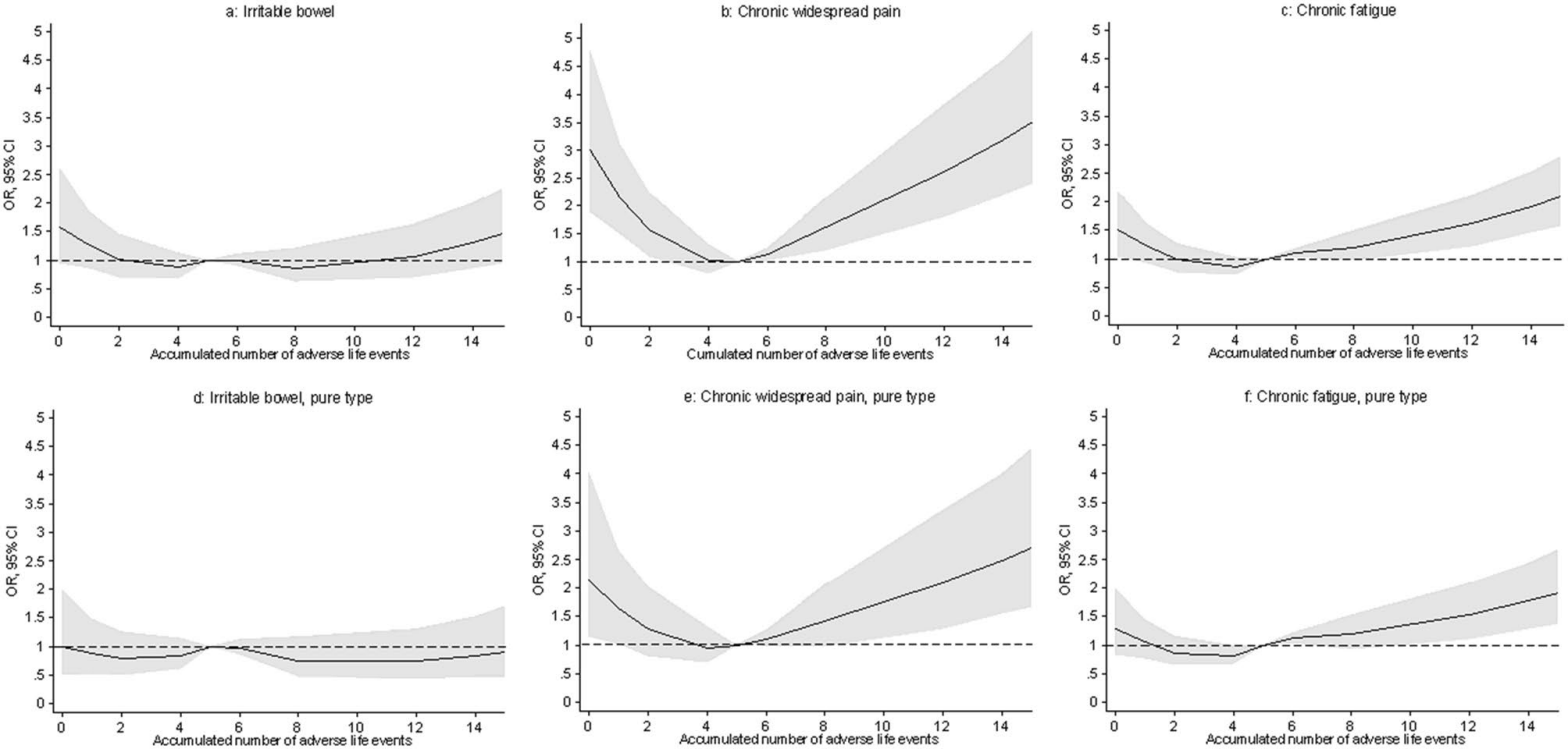
Association between the accumulated number of adverse life events and irritable bowel, chronic widespread pain, and chronic fatigue. Cases were established by means of self-reported questionnaires. Pure types constitute cases with only one syndrome, i.e. no comorbidity of one of the other syndromes. All analyses were adjusted for sex, age, personality traits, social status, and self-efficacy.

Significant positive associations with the accumulated number of ALE were found for all definitions of FSD besides from IB and IB pure, when cases were established by means of self-reported questionnaires (Figs. 1, 2, 3). Further, the association showed a J-shaped curve with a negative association for few negative life events changing into a positive association with increasing number of adverse life events.

The associations were less strong, when cases were established by means of the diagnostic interview. Here, significant associations were only found for FSD, multi-organ type (Fig. 1) and for the GS single-organ type (Fig. 2). However, the number of cases was low in these groups which reduced the possibility of adjusting for confounding variables. Hence, these analyses were only adjusted for sex.

Regardless of FSD definition and assessment method, we could not reject the hypothesis of no modification by level of self-efficacy. Hence, no modification by level of self-efficacy was found (*p* > 0.05).

### The moderating effect of having both higher neuroticism and higher accumulated number of adverse life events

We found no significant moderating effect of neuroticism and the accumulated number of ALE on the probability of having FSD. This was the case for all definitions of FSD. Graphs showing the additive effects of having higher neuroticism *and* higher number of ALE on the probability of having FSD are displayed in Figs. S1–S3 in the Supplementary Material.

## Discussion

In the present study, we found that higher level of neuroticism as well as higher accumulated number of ALE were positively associated with FSD. Hence, to our knowledge, this is the first study that confirms these associations in a highly powered unselected population-based sample. In none of the analyses could we reject the hypothesis of no modification by level of self-efficacy. Having a higher level of neuroticism and having experienced higher accumulated number of ALE did not have further impact on the probability of having FSD.

Our finding of a significant positive association between neuroticism and FSD is in accordance with several other studies from clinical settings including patients with chronic fatigue syndrome^7–10^, multi-organ BDS^11^, somatoform disorders^12^, and dissociative disorders^13^. However, our finding is in contrast to a study including a representative community-based cohort which did not find an association between FSD and neuroticism^14^. This discrepancy may be caused by methodological differences: First, different measures of neuroticism were used, and meta-analyses have suggested that effect sizes on neuroticism may differ depending on measurement used^23,24^. Second, in the study by Kingma et al.^14^, FSD diagnoses were based on participants’ self-report, i.e. participants had to indicate whether they had received a diagnosis of FSD from a pre-defined list including seven different FSD diagnoses. In the current study, however, we established FSD by means of validated case-finding instruments and diagnostic interviews.

Our finding that participants with FSD scored higher on neuroticism than participants without FSD may have different theoretical implications. A high level of neuroticism might act as a predisposing factor for FSD in the sense that individuals with a generic vulnerability may have a tendency to respond with negative emotions but also with physical arousal to external distressing events, hence, manifesting a lower threshold for the sensation and manifestation of physical symptoms^11,25,26^. On the other hand, one might assume neuroticism to be affected by state-effects such as depression and anxiety. Thus, the association between neuroticism and FSD observed in the present study may be a reflection of higher negative emotions caused by the FSD. Hence, being a consequence of FSD, the level of neuroticism might therefore change if a participant was successfully treated for the FSD^27–30^.

In the present study, we also found positive associations between the accumulated number of ALE and FSD, when cases were established by means of self-reported questionnaires. This finding is in line with other population-based studies on functional somatic syndromes^15,16,18^ and functional bodily symptoms^17^. When cases were established with diagnostic interviews, the same tendency was seen but the association only remained significant for the FSD multi-organ type and for the GS single-organ FSD type. It is, however, important to notice that the number of cases within these groups was low. Hence, it was not possible to adjust for other confounding variables than sex. Therefore, these significant associations may be explained by confounding factors such as age, social status, personality, and self-efficacy.

For most definitions of FSD, especially when cases were established by self-reported questionnaires, we found a U-shaped association with the accumulated number of ALE, indicating a resilience for individuals having experienced a low number of ALE. CLAM includes a wide range of ALE from ‘normal life events’ such as death of a grandparent to major traumas such as suffering a loss in a tragedy or disaster. The experience of the more common and “milder” ALE has been suggested to serve as a protecting factor, strengthening and building up one’s self-efficacy. However, if repeatedly experiencing more ALE, at some point, the protecting tendency will shift and the accumulated load of ALE may instead serve as risk factor for the development of mental and physical conditions^31–33^.

A systematic review and meta-analysis by Afari et al.^18^ established an association between trauma and functional somatic syndromes but argued that the format of trauma assessment method may influence how respondents fill in the questions about trauma exposure. The current study used retrospective self-report of lifetime ALE which may cause recall bias and thereby differential misclassification regarding to the report of the accumulated number of ALE. Even though the CLAM is well-validated for measuring lifetime ALE, it may be reasonable to suggest that since this scale also includes many milder ‘normal-life’/non-traumatizing events, this may bias the obtained association with FSD towards one. The interpretation and generalization of results regarding ALE should therefore be seen in the light of which scale is used for measuring the ALE and how the events are measured. The CLAM measures lifetime events. Yet, another approach could be to focus on specific events, e.g. sexual abuse^16^ or specific lifetime periods, e.g. events happened in childhood^34^, which has been done in other studies. The CLAM makes use of the typological approach by conceptualizing the included events as adverse/negative a priori. This approach has been criticized for oversimplifying individual differences. To accommodate for this, measures such as the Event Characteristic Questionnaire, which measures both event-focused and consequence-focused characteristics^35^ and thereby using a multidimensional approach, have been suggested. It takes individual differences into account by describing multiple dimensions of perceived characteristics of life events. Future studies may benefit from focusing more on the multidimensional approach and including ratings of e.g. the perceived seriousness of the event. This could be done by using an a priori weighing of each event according to its seriousness^36^, or to ask about the subjective experience of seriousness. However, conducting a large general population study as the current, including a large battery of questionnaires, we were forced to include questionnaires with reduced length and complexity to secure a higher response rate. Therefore, we made a prioritization and included the CLAM, gaining the opportunity to measure the number and time of occurrence of a wide range of adversities within one scale.

The present study has several strengths: First, it included a large sample from the general population with almost equal distribution of women and men. The population-based study design reduces the risk of selection bias and, hence, increases the generalization to other adult populations. Second, as many definitions of FSD have been proposed^1^, the current study used more than one delimitation to capture the diverse nature of FSD as both mono- and multi-systemic conditions. Third, many epidemiological studies use self-reported questionnaires only for defining FSD cases. This is a convenient and cost-effective method when studying large samples, but the validity of this method for identification of clinically relevant cases may be questionable. In the current study, FSD were based on both self-reported questionnaires and diagnostic interviews performed by trained family physicians.

The present study also has some limitations which should be addressed: First, the response rate of 29.5% for the questionnaire sample and 64.9% for the interviewed sample may be considered low. Even though the risk of selection bias was markedly reduced compared to clinical studies, we cannot rule it out completely. However, a previous study including non-responder analyses indicated that selection bias did not seem to influence noticeably on social parameters^37^. Furthermore, a previous population-based study including the Danish Health 2006 cohort found equal scores of neuroticism and self-efficacy as we did in our study^22^. Second, using primary care physicians and not psychologically or psychiatrically trained specialists for diagnosing FSD during the interviews might also be a limitation. However, all interviewers had several years of experience from primary care and they were thoroughly trained before the study by psychiatrists with long-time experience in diagnosing patients with FSD in specialized clinical settings. Hence, we do not believe that this constitutes a major limitation in our study. Third, given the cross-sectional design of the study, it cannot be definitely determined whether the findings are consequences or determinants of FSD. However, given the knowledge about development and stability of personality and the fact that the accumulated number of ALE had occurred before the DanFunD baseline investigation and thereby the establishment of FSD, we may argue that our results suggest possible relationships of causality among determinants of neuroticism and ALE for FSD.

## Conclusion

We found that neuroticism and ALE were important factors in FSD. Positive associations between FSD and neuroticism and the accumulated number of ALE, respectively, were found, and these associations were not modified by level of self-efficacy. Further, having a higher level of neuroticism as well as having experienced a higher accumulated number of ALE did not add further to the probability of having FSD. Our results underline the complexity of FSD, not only regarding their aetiology but also when managing and treating the patients within a bio-psycho-social framework. Our study contributes with important knowledge to clinicians when explaining the mechanisms behind FSD to the patient.

## Methods

### Study population

The present study is based on the Danish Study of Functional Disorders (DanFunD) part two baseline cohort^38^. Participants were randomly drawn from the Danish Civil Registration System^39^, and the exclusion criteria were as follows: not born in Denmark, not being a Danish citizen, and pregnancy.

The DanFunD part two baseline cohort comprises a total of 7493 (29.5% of invited participants) men and women aged 18–72 years, born in Denmark, and living in the Western part of greater Copenhagen. All participants filled in questionnaires regarding physical symptoms and psychological factors, among others. A stratified subsample (*n* = 2450) of every tenth participant and every high score on the DanFunD baseline symptom questionnaires was invited to participate in a diagnostic interview, the Research Interview for Functional somatic Disorders (RIFD), performed by trained family physicians^40^; 1590 accepted and participated in the interview.

### Ethics

The authors assert that all procedures contributing to this work comply with the ethical standards of the relevant national and institutional committees on human experimentation and with the Helsinki Declaration of 1975, as revised in 2008. All procedures involving human subjects were approved by the Ethical Committee of the Capital Region of Denmark (H-3-2011-081, H-3-2012-015). Written informed consent was obtained from all subjects.

### Case definition of functional somatic disorders

For operationalisation of FSD, we used the unifying diagnostic construct of Bodily Distress Syndrome^1,41,42^. It presents with four symptom clusters; a cardiopulmonary cluster, a gastrointestinal cluster, a musculoskeletal cluster, and a general symptoms/fatigue cluster. The diagnosis divides patients into a single/oligo-organ type, i.e. having symptoms from one or two of the symptom clusters, and a multi-organ type, i.e. having symptoms from at least three of the symptom clusters^42^. The diagnostic construct has been validated and confirmed in several clinical and population-based studies and has shown to encompass a range of FSS such as IB, CWP, and CF^43–46^.

In a previous paper, we have argued for the benefits of including more definitions of FSD to capture the nature of these conditions^47^. Therefore, in this paper, we present data both using the FSD diagnosis, conceptualized as Bodily Distress Syndrome, and three commonly used FSS, i.e. IB^48^, CWP^49^, and CF^50^. Due to overlap between the FSS^2^, the pure types of each syndrome, i.e. individuals with only one of the three syndromes, were included as well.

#### Assessment of FSD

Participants with FSD were identified by the self-reported Bodily Distress Syndrome Checklist^42^ including bothersome symptoms within the last 12 months. Additionally, a stratified subsample of participants with a clinical diagnosis of FSD was identified with the RIFD interview, developed to be used as a second phase tool after a respondent’s self-report in symptom questionnaires. The RIFD interviews were performed over the telephone by three trained primary care physicians with at least 12 years of practise in family medicine. Before the study, they had all been trained in conducting the RIFD interviews and diagnosing FSD by a psychiatrically trained specialist with long-time experience in diagnosing patients with FSD in specialized clinical settings. The physicians assessed whether or not a specific symptom pattern was due to an FSD or better attributable to another physical or mental condition. The RIFD interview has shown good criterion validity for identifying individuals with FSD^40^.

Participants with IB, CWP, and CF were identified with self-reported validated symptom questionnaires including bothersome symptoms within the last 12 months.

### Primary measures

#### Neuroticism

Neuroticism was measured with the Danish version of the short-form NEO Personality Inventory (NEO-PI-Rsf)^51,52^, an instrument used to measure the five personality domains (1) neuroticism, (2) extraversion, (3) openness, (4) agreeableness, and (5) conscientiousness. The NEO-PI-Rsf includes 60 self-descriptive statements such as “I often feel tense and jittery”. It is rated with a five-point rating scale from “strongly disagree” to “strongly agree”. In the present study, the domain for neuroticism was scored from 0 to 48 and a higher score indicated a higher level of neuroticism.

#### Accumulated number of adverse life events

The accumulated number of ALE was measured with the Danish version of the Cumulative Lifetime Adversity Measure (CLAM)^31^. CLAM examines the cumulative effect of a range of ALE including the number of exposures to the same event. CLAM obtains exposure to lifetime adversity by asking the respondents whether they have experienced 37 different ALE. The ALE may constitute own illness or injury, loved one’s illness or injury, violence (e.g. being physically attacked or sexually assaulted), bereavement (e.g. death of a child), social/environmental stress (e.g. experienced serious financial difficulties), relationship stress (e.g. getting a divorce), disaster (e.g. suffered loss in a major fire, flood, earthquake). CLAM also gives the possibility to add one other unnamed life event. An example of an item could be “Have you suffered from a serious illness” which is answered “yes” or “no”. Consequently, the respondents write the age at which each event occurred or an age interval if the event had occurred for a time period. A sum score is calculated by adding age time points and age ranges by simply counting age time points and age ranges, i.e. an age range counted for one event and an age time point counted for one event. Each type of ALE can maximally receive a score of four, i.e. the event could happen up to four times.

### Secondary measures

#### Self-efficacy

Self-efficacy was measured with the General Self-Efficacy Scale (GSE)^53^, a questionnaire assessing people’s beliefs in their capabilities to perform a specific action required to attain a desired outcome. A typical item in this scale is “Thanks to my resourcefulness, I can handle unforeseen situations”. The scale consists of 10 items rated on a four-point rating scale from “not at all true” to “exactly true”. A sum score is calculated, and higher scores indicate a higher degree of self-efficacy. In the present study, the sum score ranged from 0 to 30.

#### Social status

Subjective social status was measured with an item requesting the participants to rate their own social status on a scale of 1 to 10; 1 being the lowest and 10 being the highest status in society^54^.

### Statistical analyses

All analyses were performed in Stata 16.0 for Windows (StataCorp LLC, College Station, USA)^55^. Descriptive statistics were presented as means and SD or as medians and IQR depending on the distribution of the continuous variables. For categorical variables, frequencies with percentages were shown.

For the testing of hypotheses 1 and 2, we first tested for possible interactions between self-efficacy and neuroticism and the accumulated number of ALE, respectively. Then, a number of logistic regression models were conducted including the dichotomous case group as the primary outcome variable and the mental factors as primary explanatory variable. Potential confounders included in the analyses were identified using directed acyclic graphs (DAGs) constructed in the browser-based program DAGitty version 3.0^56^. For the testing of hypothesis 1, association between FSD and neuroticism, potential confounders constituted (prioritized order): sex, age, socioeconomic status, number of adverse life events, and self-efficacy. For the testing of hypothesis 2, association between FSD and accumulated number of ALE, potential confounders constituted (prioritized order): sex, age, socioeconomic status, personality, and self-efficacy. It was necessary to prioritize the confounding explanatory variables to prevent overfitting in the analyses as the number of cases in some of the primary outcome variables was low. For the adjustment of personality in the testing of hypothesis 2, we wanted to take into account any confounding effect from the whole personality spectrum and not only for neuroticism. Therefore, a principal component analysis of the five personality traits from the NEO-PI-Rsf was performed to reduce the dimensionality of the five personality traits and thereby reducing the number of variables to include as confounding factors. In the end, we chose two principal components to be included in the analyses when adjusting for personality.

The (continuous) explanatory variables were modelled using restricted cubic splines with five knots at the 5th, 27.5th, 50th, 72.5th, and 95th percentiles according to the recommendations by Harrell^57^ to avoid the strong assumption of a linear effect on the log odds of the outcome. We then tested whether there were any deviations from linearity using a χ^2^-test (*p* < 0.05).

Associations were reported as OR with 95% CI. To illustrate the effect of the continuous variables modelled by restricted cubic splines, we used the user written Stata command xbrcspline^55^.

For the testing of hypothesis 3, a moderation analysis including an interaction term between neuroticism and the accumulated number of ALE to the logistic regression model was performed. Interactions were presented graphically by showing the probability of FSD as a function of the accumulated number of adverse life events at the 10%, 25%, 50%, 75%, and 90% percentiles of neuroticism.

## Supplementary Information

Supplementary Figures.

## Data Availability

The research plan and the datasets generated during and/or analyzed during the current study are available from the corresponding author on reasonable request.
