# Supplementary material for: Neuroticism and adverse life events are important determinants in functional somatic disorders: the DanFunD study
Source: Sci Rep. 2022 Nov 15;12:19604. doi: 10.1038/s41598-022-24213-6 (PMC9666664; doi:10.1038/s41598-022-24213-6)
Supplement: Supplementary file 1 — Supplementary Figures. [file 41598_2022_24213_MOESM1_ESM.pdf]

Supplementary information

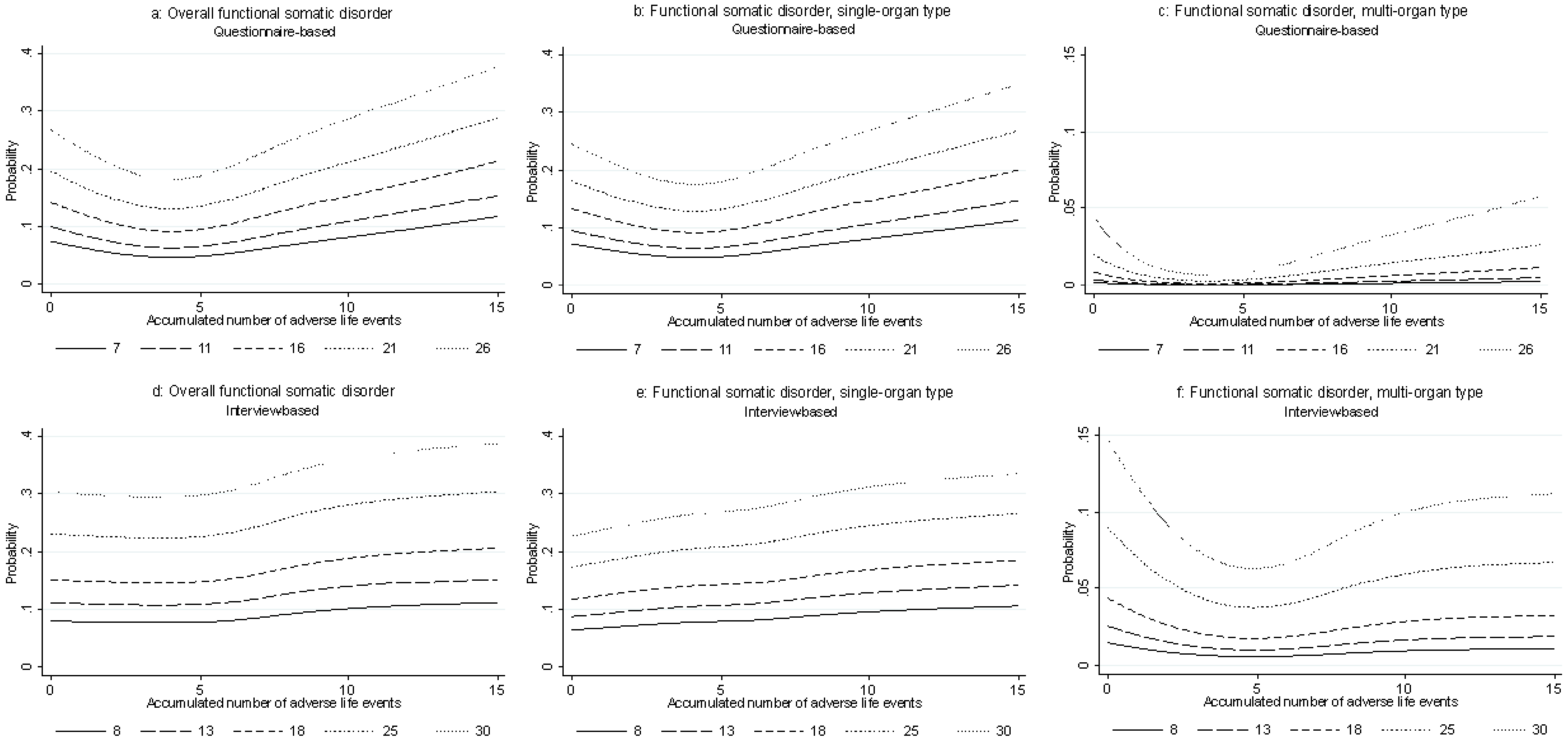

Figure S1: Association between the accumulated number of adverse life events and functional somatic disorder at different levels of neuroticism (levels are indicated with different lines). Cases are established by means of self-reported questionnaires and diagnostic interviews. a, b, d, and e were adjusted for sex, age, social status, personality, and self-efficacy. c and f were only adjusted for sex because of low number of cases.
NB! The probability scales for the multi-organ type, (c and f) have different ranges than the probability scales for the other types.

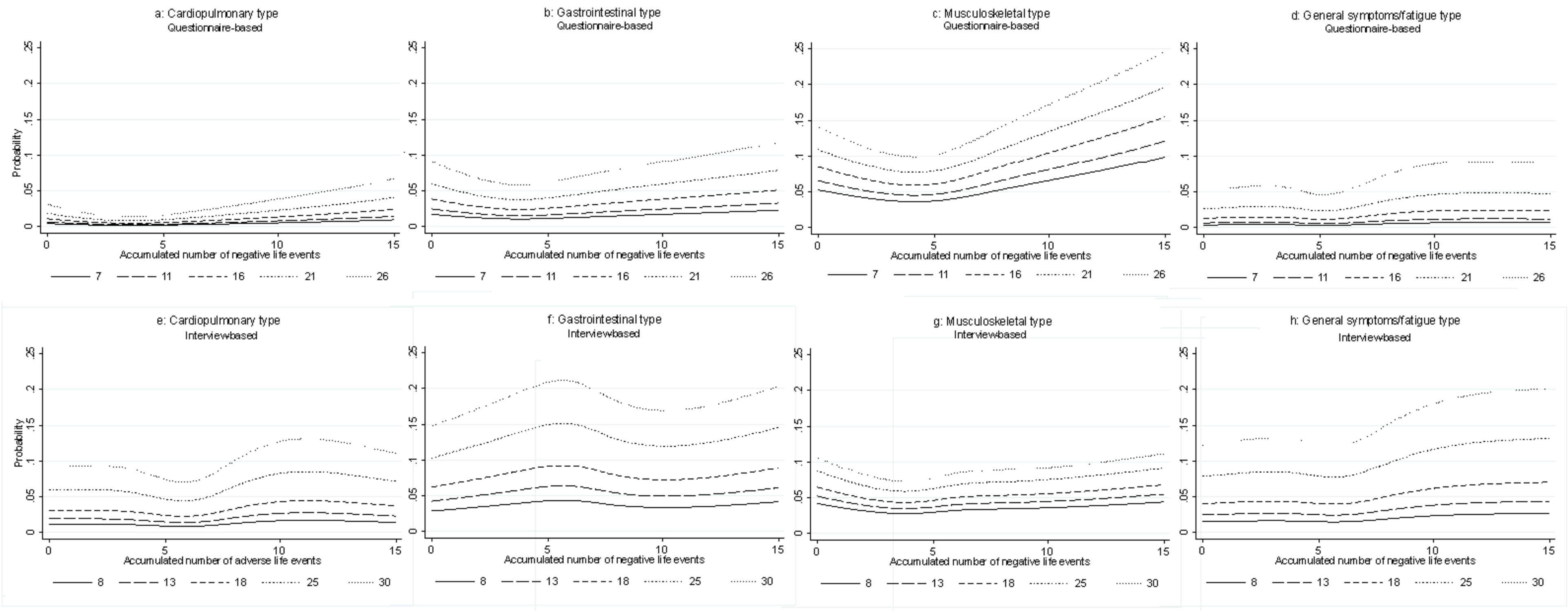

Figure S2: Association between the accumulated number of adverse life events and single-organ types of functional somatic disorders at different levels of neuroticism (levels are indicated with different lines). Cases are established by means of self-reported questionnaires and diagnostic interviews. a-d and f were adjusted for sex, age, social status, personality, and self-efficacy; e was unadjusted; g and h were adjusted for sex and age.

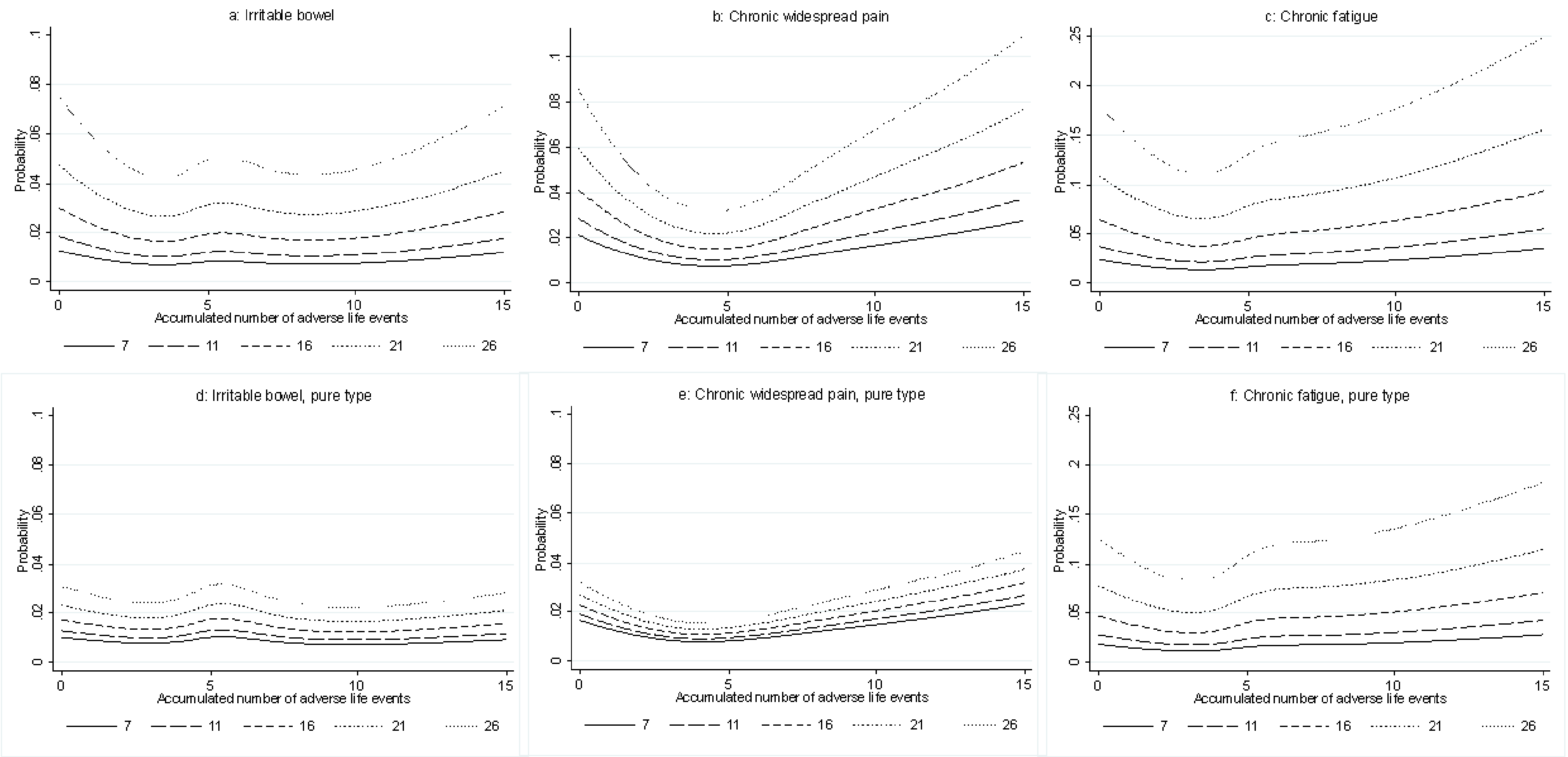

Figure S3: Association between the accumulated number of adverse life events and irritable bowel, chronic widespread pain, and chronic fatigue at different levels of neuroticism (levels are indicated with different lines). Cases are established by means of self-reported questionnaires. Pure types constitute cases with only one syndrome, i.e. no comorbidity of one of the other syndromes. Analyses were adjusted for sex, age, social status, personality, and self-efficacy.

NB! The probability scales for chronic fatigue and chronic fatigue, pure type, have different ranges than the probability scales for irritable bowel and chronic widespread pain.
